# Designing and Engineering *Methylorubrum extorquens* AM1 for Itaconic Acid Production

**DOI:** 10.3389/fmicb.2019.01027

**Published:** 2019-05-09

**Authors:** Chee Kent Lim, Juan C. Villada, Annie Chalifour, Maria F. Duran, Hongyuan Lu, Patrick K. H. Lee

**Affiliations:** ^1^School of Energy and Environment, City University of Hong Kong, Hong Kong, China; ^2^Department of Chemistry, City University of Hong Kong, Hong Kong, China

**Keywords:** itaconic acid, *Methylorubrum extorquens* AM1, poly-β-hydroxybutyrate, *phaR*, methanol, transcriptomic, metabolic engineering

## Abstract

*Methylorubrum extorquens* (formerly *Methylobacterium extorquens*) AM1 is a methylotrophic bacterium with a versatile lifestyle. Various carbon sources including acetate, succinate and methanol are utilized by *M. extorquens* AM1 with the latter being a promising inexpensive substrate for use in the biotechnology industry. Itaconic acid (ITA) is a high-value building block widely used in various industries. Given that no wildtype methylotrophic bacteria are able to utilize methanol to produce ITA, we tested the potential of *M. extorquens* AM1 as an engineered host for this purpose. In this study, we successfully engineered *M. extorquens* AM1 to express a heterologous codon-optimized gene encoding *cis-*aconitic acid decarboxylase. The engineered strain produced ITA using acetate, succinate and methanol as the carbon feedstock. The highest ITA titer in batch culture with methanol as the carbon source was 31.6 ± 5.5 mg/L, while the titer and productivity were 5.4 ± 0.2 mg/L and 0.056 ± 0.002 mg/L/h, respectively, in a scaled-up fed-batch bioreactor under 60% dissolved oxygen saturation. We attempted to enhance the carbon flux toward ITA production by impeding poly-β-hydroxybutyrate accumulation, which is used as carbon and energy storage, via mutation of the regulator gene *phaR*. Unexpectedly, ITA production by the *phaR* mutant strain was not higher even though poly-β-hydroxybutyrate concentration was lower. Genome-wide transcriptomic analysis revealed that *phaR* mutation in the ITA-producing strain led to complex rewiring of gene transcription, which might result in a reduced carbon flux toward ITA production. Besides poly-β-hydroxybutyrate metabolism, we found evidence that PhaR might regulate the transcription of many other genes including those encoding other regulatory proteins, methanol dehydrogenases, formate dehydrogenases, malate:quinone oxidoreductase, and those synthesizing pyrroloquinoline quinone and thiamine co-factors. Overall, *M. extorquens* AM1 was successfully engineered to produce ITA using acetate, succinate and methanol as feedstock, further supporting this bacterium as a feasible host for use in the biotechnology industry. This study showed that PhaR could have a broader regulatory role than previously anticipated, and increased our knowledge of this regulator and its influence on the physiology of *M. extorquens* AM1.

## Introduction

Itaconic acid (ITA), a C-5 dicarboxylic organic acid, is used as a polymer building block and is listed as one of the top 12 value-added chemicals produced from biomass by the United States Department of Energy (Werpy et al., 2004).

The polymers derived from ITA have broad industrial uses including as ingredients for making superabsorbent polymers, as co-builders in detergents, as mineral dispersants in paint coating, as anti-scaling agents in water treatment processes, and as sizing agents for carpets (Okabe et al., 2009; Klement and Buchs, 2013).

The current commercial production of ITA is by fermentation with *Aspergillus terreus*, but this process is expensive due to the requirement for feeding sugars as substrates, as well as other undesirable characteristics in cultivation including spore formation, susceptibility to damage by shear stress and filamentous growth (Jeon et al., 2016). To circumvent these issues, several bacterial hosts including *Escherichia coli* (Chang et al., 2017), *Corynebacterium glutamicum* (Otten et al., 2015), and *Synechocystis* sp. (Chin et al., 2015) have been examined for ITA production.

To date, no attempt has yet been made to develop a bacterial host that can utilize methanol to produce ITA. Methanol is a promising low-cost renewable feedstock whose production does not compete with food supply, and it is a substrate with low biotic contamination risk during fermentation (Schada von Borzyskowski et al., 2018), which has been used as a feedstock for bioproduction of a variety of value-added compounds (Zhang et al., 2018). The α-proteobacterium *Methylorubrum extorquens* (formerly *Methylobacterium extorquens*) AM1 (hereafter referred to as AM1) is a versatile methylotrophic bacterium that utilizes a variety of carbon substrates including C-1 molecules such as methanol, methylamine and formate, and multi-carbon molecules such as pyruvate, succinate, lactate, and acetate (Green and Ardley, 2018). Previously, AM1 has been engineered to produce various value-added compounds including polyhydroxyalkanoate terpolymer (Orita et al., 2014), mevalonic acid (Zhu et al., 2016), mesaconic acid (Sonntag et al., 2015), methylsuccinic acid (Sonntag et al., 2015), and crotonic acid (Schada von Borzyskowski et al., 2018).

In this study, we tested the feasibility of AM1 as an engineered host for ITA production. AM1 was successfully engineered to produce ITA using acetate, succinate and methanol as substrates. We further designed and tested a *phaR* mutant derivative strain, deficient in PHB accumulation, for potentially more efficient ITA production. PHB is a class of PHA that serves as a carbon and energy store in microorganisms. Transcriptomic analysis was performed to understand the global gene expression profiles during ITA production of the engineered strains. Finally, scaled-up production of ITA in fed-batch bioreactors was investigated. This study provided insights into the engineering of methylotrophic hosts to produce ITA from the promising alternative feedstock methanol.

## Materials and Methods

### Culture Conditions

*Escherichia coli* strains were cultivated in Luria-Bertani medium at 37°C. *M. extorquens* AM1 (ATCC 14718) was purchased from the American Type Culture Collection (ATCC). AM1 was routinely grown in the minimal medium adapted from Zhu et al. (2016) (MC, Supplementary Table S1) as 25 mL culture in 125 mL-serum bottles containing 124 mM methanol at 30°C with shaking at 200 rpm. The bottles were loosely capped to allow exchange of atmospheric gases. Other minimal media utilized were adapted from Mokhtari-Hosseini et al. (2009) (HM), Choi et al. (1989) (CM), and MM (Supplementary Table S1). The inoculum from a 2-day-old culture was added to 50 mL medium to an OD_600_ of 0.02 at the start of an experiment where sodium acetate (5 or 30 mM), disodium succinate (15 mM) or methanol (240 mM) was used as a carbon source. Triplicate biological cultures were prepared for batch experiments. Antibiotics at the following concentrations were used when required for selective culture: kanamycin at 50 μg/mL for both *E. coli* and AM1; ampicillin at 100 μg/mL for *E. coli*; tetracycline at 15 μg/mL, and 10–20 μg/mL for *E. coli* and AM1, respectively. Cell culture OD_600_ was measured using a spectrophotometer (SpectraMax M2e, Molecular Devices, United States). Strains used in this study are listed in Table 1.

**Table 1:** Strains and plasmids used in study.

Strain	Genotype	Source
*M. extorquens* AM1	Wildtype	ATCC, United States
WT_101	*M. extorquens* AM1 carrying pTE101 plasmid	This study
WT_CAD	*M. extorquens* AM1 carrying pTE101a-CAD plasmid	This study
Δ*phaR*	*M. extorquens* AM1 with *phaR* gene truncated from nucleotide position of 269 to 599	This study
Δ*phaR*_CAD	Δ*phaR* strain carrying pTE101a-CAD plasmid	This study
*E. coli* S17-1λ*pir*	*recA thi pro*, *tra* genes (from plasmid RP4) integrated into chromosome, λ*pir* lysogen	Simon et al. (1983)
*E. coli* TOP10	F- *mcrA* (*mrr-hsdRMS-mcrBC*) *80lacZM15 lacX74* *recA1 ara139* (*ara-leu*) *7697 galU galK rpsL* (Str^R^) *endA1 nupG*	Invitrogen, United States

**Plasmid**	**Characteristic**	**Source**

pUC57-CAD	Chemically synthesized codon optimized *cad* gene in pUC57 vector, Ap^R^	Biomatik, United States
pMiniT	Linearized cloning vector, Ap^R^	New England BioLabs, United States
pTE101	Brick vector, no promoter, Km^R^	Schada von Borzyskowski et al. (2015); Addgene, United States
pTE102	Brick vector, *mxaF* promoter (pMxaF), Tc^R^	Schada von Borzyskowski et al. (2015); Addgene, United States
pTE102-CAD	pTE102 plasmid with *cad* gene inserted downstream of a ribosomal binding site	This study
pTE101a	pTE101 plasmid containing pMxaF	This study
pTE101a-CAD	pTE101a plasmid with pMxaF: *cad*	This study
pCM433	*sacB*-based allelic exchange vector, Ap^R^ Cm^R^ Tc^R^	Marx (2008); Addgene, United States
p433-phaR-UD	pCM433 plasmid containing upstream and downstream DNA regions of *phaR* gene section, for creating the Δ*phaR* strain	This study


### Metabolic Engineering of AM1 Strains

Plasmids and sequences of the primers used in this study are listed in Table 1 and Supplementary Table S2, respectively. The gene encoding *cis-*aconitic acid decarboxylase (*cad*) utilized in this study was based on the amino acid sequence of the enzyme from *A. terreus* (GenBank accession no. BAG49047.1) and was codon optimized using the Codon Optimization OnLine (COOL) software (Chin et al., 2014) according to the codon usage of 184 genes that were deemed significantly expressed in AM1 (Laukel et al., 2004; Bosch et al., 2008; Schneider et al., 2012a,b) (Supplementary Tables S3, S4). Molecular cloning work was performed with *E. coli* TOP10. The *cad* gene was excised from pUC57-CAD with *Vsp*I and *Hind*III, and was subsequently ligated into the pTE102 plasmid at the same restriction sites, creating pTE102-CAD where the *cad* gene was downstream of a ribosomal binding site sequence which worked efficiently in AM1 (Schada von Borzyskowski et al., 2015). Separately, the pMxaF promoter region from pTE102 was excised with *Bgl*II and *EcoR*I, and ligated into pTE101 at the same restriction sites to create pTE101a. Subsequently, the *cad* assembly from pTE102-CAD was excised using *Xba*I and *Pst*I and inserted downstream of the pMxaF promoter in the pTE101a via ligation at the *Spe*I and *Pst*I sites to create pTE101a-CAD. This final expression construct was electroporated into AM1 according to the protocol of Toyama et al. (1998).

To create the *phaR* mutation with in-frame truncation (by excising 339 bp out of the entire gene length of 612 bp), a DNA region was amplified by the phaR_Up-F and phaR_Up-R primers from the AM1 gDNA and cloned into pMiniT (New England BioLabs, United States). A fragment of this construct was excised with *Xho*I and *Pst*I and cloned into pCM433 (Marx, 2008) at the same restriction sites. After that, the PCR product amplified from gDNA using the phaR_Down-F and phaR_Down-R primers was directly cloned into the pCM433-based construct above at the *Pst*I and *Vsp*I sites, creating the allelic exchange plasmid p433-phaR-UD. This plasmid was conjugated into AM1 by *E. coli* S17-1 λ*pir* using the method adapted from Chistoserdova and Lidstrom (1994). Mutant colonies (i.e., Δ*phaR* strain) were screened for sensitivity to tetracycline and verified by PCR.

PCR amplification was performed with the Accura High-Fidelity Polymerase (Lucigen, United States), while restriction enzymes and T4 DNA ligase were purchased from New England BioLabs (United States) and Promega (United States).

### Analytical Measurements

Itaconic acid, acetate, and methanol were measured using liquid chromatography equipped with a photodiode array detector (210 nm wavelength) and a refractive index detector (35°C) (ACQUITY UPLC, Waters Corporation, United States). The Aminex Ion Exclusion HPX-87H column (65°C) (Bio-Rad, United States) with 5 mM H_2_SO_4_ mobile phase (0.4 mL/min) was used.

Poly-β-hydroxybutyric acid analysis was adapted from Taguchi et al. (2001). Briefly, the cell pellet was first dried overnight at 55°C. Concentrated H_2_SO_4_ (1 mL) was added to the sample and boiled at 120°C for 40 min. Subsequently, 4 mL of 7 mM H_2_SO_4_ was added and the solution was filtered before liquid chromatography analysis with the same conditions as above except 7 mM H_2_SO_4_ was used as the mobile phase. PHB standards (Sigma-Aldrich, United States) were subjected to the same treatment as the samples.

### RNA Extraction and Sequencing

For each sample for RNA-Seq, three bottles of culture grown in 50 mL HM medium containing 240 mM methanol and kanamycin (OD_600_ = 0.5–0.6) were pooled and 100 mL was used for RNA extraction. The cells were pelleted by centrifugation at 10,000 rpm for 10 min, snap frozen with liquid nitrogen and immediately stored at -80°C. Total RNA was extracted using the RNeasy Mini kit (Qiagen, Germany) after cell lysis with lysozyme (7.5 mg/mL) (Sigma-Aldrich, United States) for 10 min, followed by homogenization using the Mini-Beadbeater-16 (BioSpec Products, United States) with autoclaved 0.1 mm zirconia/silica beads (BioSpec Products, United States) for 5 min. gDNA removal with DNase (Qiagen, Germany) was performed according to the manufacturer’s instructions. rRNA removal was performed using the Ribo-Zero Magnetic Kit (Illumina, United States) and the rRNA-depleted RNA samples were used as templates to create cDNA libraries containing 250–300 bp inserts with the NEBNext Ultra Directional RNA Library Prep Kit for Illumina sequencing (New England BioLabs, United States). Paired-end sequencing (150 bp) was performed using the Illumina HiSeq 4000 sequencing platform, generating about 1 Gb of raw data per sample. Three biological replicates of each strain were prepared for RNA-Seq.

### Analysis of RNA-Seq Data

Raw sequencing reads were subjected to quality control using FastQC v0.11.5 (Andrews, 2010) and illumina-utils v2.0.2 (Eren et al., 2013) following best practice criteria for RNA-Seq analysis (Conesa et al., 2016). High-quality reads were pseudo-aligned to the AM1 gene sequences (GCF_000022685.1) using kallisto v0.43.1 (Bray et al., 2016) with 100 bootstraps per sample. Differential expression was analyzed using sleuth v0.29.0 (Pimentel et al., 2017) with integration of bootstraps from the pseudo-alignment. Transcript level was reported as Transcripts Per Million (TPM). The Wald test was used to assess the differential expression of transcripts and the transformation function log_2_(x + 0.5) (Sahraeian et al., 2017) was passed to sleuth quantification to calculate the effect size (β value) as log_2_-based fold changes. Log_2_-based fold changes of less than -1 or greater than 1, in conjunction with a false discovery rate-adjusted *p*-value <0.01, were used as the threshold for identifying significant differential gene expression (Liu et al., 2018). Gene expression profiles were analyzed by comparing WT_CAD against WT_101, Δ*phaR*_CAD against WT_101 and Δ*phaR*_CAD against WT_CAD. Unless otherwise indicated, all the genes described were significantly differential expressed. KEGG Orthology assignment was made using BlastKOALA (Kanehisa et al., 2016) and gene ontology was obtained with eggNOG-mapper (Huerta-Cepas et al., 2017). Gene locus tags are based on the following RefSeq sequences (Vuilleumier et al., 2009): *M. extorquens* AM1 chromosome (NC_012808.1), and the four plasmids of *M. extorquens* AM1 [megaplasmid (NC_012811.1), p1META1 (NC_012807.1), p2META1 (NC_012809.1), and p3META1 (NC_012810.1)].

### Bioreactor Experiments

A twin 2 L Biostat B stirred tank bioreactor (Sartorius Stedim, France) was used. As inoculum, 100 mL seed culture grown in HM with 124 mM methanol and kanamycin for 3 days was transferred into the bioreactor vessel containing 1 L of HM with 240 mM methanol and kanamycin, resulting in an initial OD_600_ of ∼0.1. After 24 h of cultivation, 2.5 or 5 mL pure methanol was added periodically using a variable-speed peristaltic pump to achieve a target methanol concentration of 240 mM. Antifoam C Emulsion (Sigma-Aldrich, United States) was added manually when necessary to prevent excessive foam formation. The incubation temperature of 30°C was maintained with a water jacket, while a pH of 7.0 was maintained with either 1 M ammonium hydroxide (NH_4_OH) or 1 M sodium hydroxide (NaOH). Dissolved oxygen concentration was maintained by the variable impeller (200–700 rpm) and compressed air (up to 1 L/min). Bioreactor experiments were performed in duplicate for each condition.

## Results and Discussion

### Design and Construction of AM1 Strains for ITA Production

AM1 is a suitable platform for ITA production as this strain is highly tolerant to the inhibitory effect of ITA (10 mM), which inhibits isocitrate lyase (Bellion and Kelley, 1979). AM1 lacks isocitrate lyase, which is used by many other bacterial species for the glyoxylate cycle, but instead employs the EMC pathway for glyoxylate regeneration (Peyraud et al., 2009). AM1 also does not utilize ITA as a carbon source (Knief et al., 2010).

**FIGURE 1:**
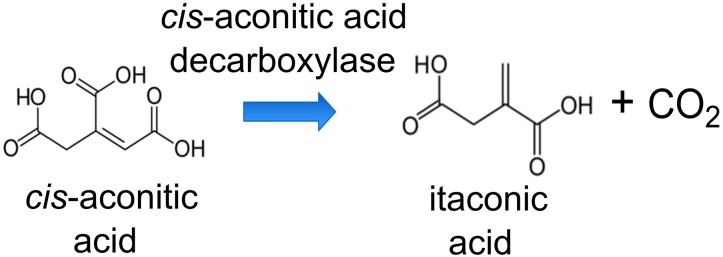
Reaction scheme of the conversion of *cis*-aconitic acid to itaconic acid (ITA) by *cis-*aconitic acid decarboxylase (Cad).

To produce ITA by AM1, we engineered the wildtype to express a heterologous codon-optimized gene encoding Cad from *A. terreus* that converts *cis*-aconitic acid to ITA (Figure 1). The *cad*-encoding gene was controlled by the pMxaF constitutive promoter in the high-copy number pTE101-based plasmid. In AM1, PHB granules can make up as much as 42% of cell dry mass (Korotkova and Lidstrom, 2001). Sonntag et al. (2015) successfully increased the production of EMC pathway-derived dicarboxylic acids by knocking out the gene encoding polyhydroxyalkanoate synthase (*phaC*), a key enzyme in PHB accumulation, and thus directing carbon flux away from storage. However, the AM1 *phaC* mutant phenotype is highly unstable (Korotkova and Lidstrom, 2001; Sonntag et al., 2015), rendering this strain not suitable as a host for our work here. Korotkova et al. (2002) investigated the regulatory role of the *phaR* gene in AM1 PHB metabolism, and found that PhaR regulates PHB biosynthesis and is involved in acetyl-CoA flux partitioning. Van Dien et al. (2003) further reported that an increase in the acetyl-CoA flux through the TCA cycle occurred when the *phaR* mutant was provided with methanol as a substrate. Taking advantage of the regulatory characteristics of PhaR, we engineered a *phaR* gene truncation mutant (Δ*phaR*) in an attempt to minimize PHB accumulation and direct more carbon toward the TCA cycle as precursors for ITA production.

### ITA Production by Engineered Strains of AM1

**FIGURE 2:**
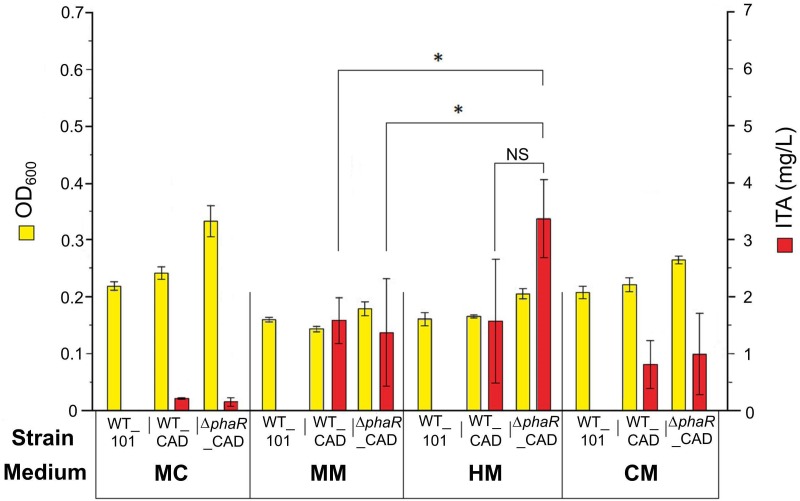
Comparison of cell biomass concentration (OD_600_) and ITA production between strains grown in different media. 5 mM sodium acetate was used as the carbon source. Samples were taken at the end of the cultivation period (day 8) after acetate was completely consumed. Low PHB content (<1%) was observed in all strains (data not shown). Asterisk (^∗^) denotes significant difference (Student’s *t*-test, *p* < 0.05), whereas NS denotes no statistically significant difference. Error bars represent one standard deviation.

The engineered strains were first tested with acetate as the carbon source, as this has been shown to promote a higher flux through the TCA cycle (Schneider et al., 2012a). However, relatively low concentrations of ITA were detected in the medium (0.22 ± 0.01 mg/L) after 5 mM acetate was consumed by WT_CAD grown in the routinely used medium MC (Figure 2). Similarly, the Δ*phaR*_CAD strain only produced small amounts of ITA (0.15 ± 0.07 mg/L) (Figure 2). We tested three other media (MM, HM, and CM) and found the HM medium was the best for ITA production, with 1.6 ± 1.1 and 3.4 ± 0.7 mg/L of ITA produced by WT_CAD and Δ*phaR*_CAD, respectively (Figure 2). This test showed that medium composition has a strong effect on ITA production by the strains. One element likely of importance is iron (FeSO_4_.7H_2_O), which is highest in the HM medium (20 mg/L), followed by MM (10 mg/L), MC (5 mg/L), and CM (1.3 mg/L) (Supplementary Table S1). The reason could be that aconitase, the enzyme responsible for producing the ITA precursor *cis*-aconitic acid, is dependent on iron for its catalytic activity (Miller and Auerbuch, 2015). The HM medium was used for subsequent experiments.

**FIGURE 3:**
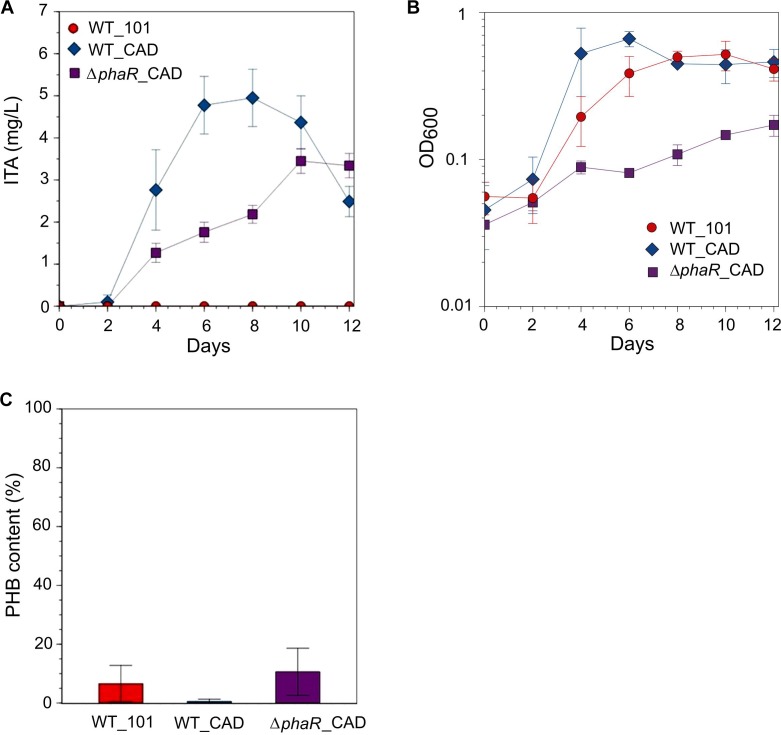
Strains grown in HM medium supplemented with 30 mM sodium acetate as the carbon source. **(A)** ITA concentration, **(B)** OD_600_ and **(C)** PHB content (PHB weight/cell dry weight) was measured on day 12 after acetate was mostly consumed. A statistically significant difference (Student’s *t*-test, *p* < 0.05) was observed in peak ITA concentration between WT_CAD (day 8) and Δ*phaR*_CAD (day 10). No statistical significant difference (Student’s *t*-test) was observed in PHB content between the strains. Error bars represent one standard deviation.

In an attempt to increase ITA titer, culturing with 30 mM acetate was tested [although the growth rate of AM1 has been reported to be reduced at this concentration (Schneider et al., 2012a)], resulting in an ITA concentration of 4.9 ± 0.7 mg/L at its peak, approximately 1.4-fold higher than when 5 mM acetate was used (Figure 3A). However, at 30 mM acetate concentration the growth of Δ*phaR*_CAD was markedly inhibited compared to both WT_101 and WT_CAD (Figure 3B). All the strains consumed most or all of the acetate in the media. Surprisingly, we observed a declining ITA concentration in the later stage of cultivation in both engineered strains (Figure 3A). AM1 cannot use ITA as the sole carbon source (Knief et al., 2010) and lacks the dedicated ITA degradation pathway found in species such as *Yersinia pestis* and *Pseudomonas aeruginosa* (Sasikaran et al., 2014). One possible reason for the gradual reduction in ITA concentration could be caused by succinyl-CoA synthetase (SucCD), which reportedly can convert ITA to itaconyl-CoA due to the structural similarity of ITA and succinic acid (Schurmann et al., 2011). AM1 provided with acetate in lieu of methanol has been shown to have elevated protein subunits of SucCD (Schneider et al., 2012a). The different strains accumulated varying amounts of PHB, including Δ*phaR*_CAD where the level observed was similar to WT_101 (Figure 3C). Korotkova et al. (2002) reported that an AM1 *phaR* mutant could still accumulate wildtype levels of PHB when grown on C-2 compounds.

Succinate (15 mM) as the sole carbon source was also tested (batch culture grown in the HM medium for 12 days), but low ITA titer was obtained, reaching a peak of 0.42 ± 0.09 mg/L for WT_CAD and 0.46 ± 0.09 mg/L for Δ*phaR*_CAD by day 8, while none was detected for WT_101. This result could be attributed to the entry point of succinate in the TCA cycle being downstream of the aconitic metabolic pathway, which is responsible for producing *cis*-aconitatic acid (the precursor for ITA).

**FIGURE 4:**
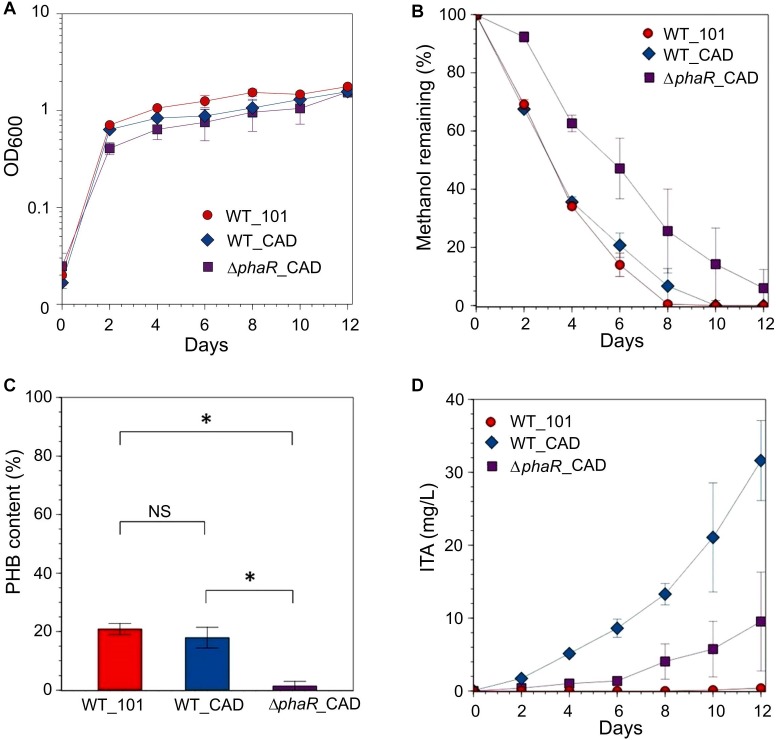
Strains grown in HM medium supplemented with 240 mM methanol as the carbon source. **(A)** OD_600_, **(B)** methanol remaining, **(C)** PHB content (PHB weight/cell dry weight) measured at the final time point and **(D)** ITA concentration. Significant differences (Student’s *t*-test, *p* < 0.05) were observed in ITA concentration between WT_CAD and Δ*phaR*_CAD at the final time point (day 12). Asterisk (^∗^) denotes statistically significant difference (Student’s *t*-test, *p* < 0.05), whereas NS denotes no statistically significant difference. Error bars represent one standard deviation.

Given the inhibitory nature of high acetate concentrations and the reduction in ITA concentration during the later stage of cultivation when acetate was the substrate, and the low concentration of ITA obtained with succinate, methanol was used as the carbon substrate in our subsequent investigations. When provided with 240 mM methanol, growth during the exponential stage and methanol consumption by Δ*phaR*_CAD were slower (growth rate: 0.0593 ± 0.007 h^-1^; doubling time: 11.8 ± 1.25 h, Supplementary Table S5) than both WT_101 (growth rate: 0.0753 ± 0.004 h^-1^; doubling time: 9.31 ± 0.56 h) and WT_CAD (growth rate: 0.0760 ± 0.002 h^-1^; doubling time: 9.12 ± 0.27 h) (Figure 4A,B). This result is similar to the AM1 *phaR* mutant growth profiles reported by Korotkova et al. (2002) and Van Dien et al. (2003) where the mutant has a lower growth rate than the wildtype strain. The considerably abundant PHB at the later stage in both WT_101 and WT_CAD (Figure 4C) might have provided them with a carbon and energy reserve compound to sustain minor growth despite having completely consumed the methanol in the medium (Handrick et al., 2000; Ratcliff et al., 2008). A substantial improvement in the ITA titer to 31.6 ± 5.5 mg/L was obtained for WT_CAD (Figure 4D), but the ITA titer of Δ*phaR*_CAD was unexpectedly lower (9.5 ± 6.8 mg/L) despite minimal PHB accumulation (Figure 4C). No reduction in ITA concentration toward the end of cultivation was observed for any strain. The lack of an increase in production of a value-added compound of interest following disruption of the PHB biosynthesis pathway has also been observed in the case of methyl ketone production by PHB-negative *Ralstonia eutropha* strains (Muller et al., 2013). The highest ITA titer achieved in batch cultures in this study was comparable to the engineered *Synechocystis* sp. PCC6803 (Chin et al., 2015), but lagged behind engineered strains of *E. coli* (Jeon et al., 2016; Chang et al., 2017) and *C. glutamicum* (Otten et al., 2015) (see Supplementary Table S6 for the comprehensive list).

### Overview of the Engineered Δ*phaR*_CAD Transcriptome

We expected a higher ITA production from Δ*phaR_*CAD relative to WT_CAD. RNA-Seq was applied to understand the underlying reasons for the lower ITA production and growth of Δ*phaR*_CAD (see Supplementary Table S7 and Supplementary Figure S1 for the expression level of each coding sequence and clustering of transcriptomic data of the samples, respectively). Unless otherwise indicated, the described gene expression for Δ*phaR*_CAD was significantly different than both WT_101 and WT_CAD.

**FIGURE 5:**
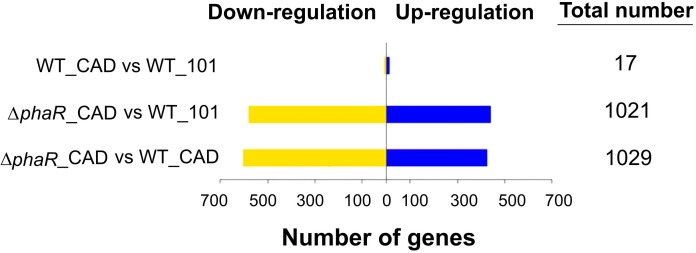
The number of up- and down-regulated genes in pairwise comparison between the strains.

The *cad* gene was highly expressed in both WT_CAD and Δ*phaR*_CAD, with less than a 1.3-fold difference in transcript level between Δ*phaR*_CAD and WT_CAD (Supplementary Table S7), suggesting that the lower ITA titer of Δ*phaR*_CAD was not due to lower expression of *cad*. A total of 17 genes were differentially expressed when comparing WT_101 and WT_CAD (Figure 5), likely resulting from the exposure of WT_CAD to ITA. Transcriptional response was also observed for *E. coli* when challenged with ITA (Rau et al., 2016). By contrast, we observed drastic changes in the transcriptome of Δ*phaR*_CAD when compared against WT_101, with 439 genes down-regulated and 582 genes up-regulated (Figure 5). Similarly, a large number of genes were differentially expressed in Δ*phaR*_CAD relative to WT_CAD, with 603 genes down-regulated and 426 genes up-regulated (Figure 5). However, relatively few genes (*n* = 5) were differentially expressed in both Δ*phaR*_CAD and WT_CAD when compared against WT_101 (Supplementary Figure S2). Given the significant transcriptional changes observed in Δ*phaR*_CAD, PhaR might have a broad regulatory role in AM1 (Supplementary Table S8), which would be consistent with reports on other species. For example, in *Rhizobium etli*, extensive proteome changes occurred when the homologous *phaR* gene was disrupted (Encarnacion et al., 2002), and the PhaR in *Bradyrhizobium diazoefficiens* has been shown to have a broad regulon extending beyond PHB metabolism (Quelas et al., 2016; Nishihata et al., 2018).

### Transcription of Genes Encoding Proteins With Regulatory Roles

The extensive transcriptomic changes observed in Δ*phaR_*CAD may be due to the differential expression of a large number of genes encoding products with regulatory roles. Among the genes that were either up- or down-regulated are those involved in general stress response including *phyR* (Gourion et al., 2008) (compared to WT_101) and two *nepR* homologs (Francez-Charlot et al., 2016) (Supplementary Table S9), where PhyR regulates 246 targets in AM1 (Gourion et al., 2008). Although we did not examine sRNA transcripts in this study, in other bacterial species PhaR may regulate the expression of sRNAs that themselves regulate cellular metabolism. For example, in *Sinorhizobium meliloti*, a PhaR homolog (AniA) controls the expression of the sRNA gene *mmgR* (Borella et al., 2017). We also observed up-regulation of the gene encoding the RNA-binding protein Hfq in Δ*phaR*_CAD (Supplementary Table S9). Hfq is involved in global post-transcriptional regulation by mediating the binding of certain sRNAs to their target mRNAs, affecting the stability and translational efficiency of the target mRNAs (Kavita et al., 2018).

Interestingly, a gene (MEXAM1_RS24770) encoding a protein which is homologous to protein acetyltransferase (Pat) of *Salmonella enterica* [39% amino acids (aa) identity] was down-regulated in Δ*phaR*_CAD compared to WT_CAD (Supplementary Table S9). In *S. enterica*, Pat is able to regulate the activities of other enzymes by acetylating their lysine sites (Wang et al., 2010). Examples of metabolic enzymes regulated in this manner include glyceraldehyde phosphate dehydrogenase, isocitrate lyase and isocitrate dehydrogenase (ICDH) kinase/phosphatase (Wang et al., 2010). In addition, transcriptional machinery such as the global transcription factor RcsB, which controls cell division, capsule biosynthesis, flagellum synthesis and chemotaxis, can be regulated by Pat via acetylation of RscB at lysine residue 180 (Thao et al., 2010). In AM1, a gene (MEXAM1_RS30515) is annotated as encoding for a transcription factor homologous to *E. coli* RscB (31% aa identity), with the corresponding Pat-specific acetylation site of RscB conserved in the homolog. Thus, reduced acetylation of the RcsB homolog by Pat in Δ*phaR*_CAD might affect its regulatory activity and explain some of the gene expression patterns observed, such as increased expression of genes related to flagellum synthesis and chemotaxis (Supplementary Table S8).

### Transcription of Genes Related to PHB Metabolism

In the study on *phaR* mutant strains reported by Korotkova et al. (2002), the transcription of several genes related to the PHB cycle was investigated, including *phaA* (encoding β-ketothiolase) and *phaB* (encoding NADPH-linked acetoacetyl-CoA reductase), which form part of the EMC pathway leading toward the PHB cycle, and *phaC* (encoding PHB synthase). Their findings differed from the transcriptomic results reported here. Korotkova et al. (2002) observed that the *phaR* mutant had increased *phaA*, *phaB*, and *phaC* gene expression after being induced with methanol, but no difference in expression of these genes was observed in this study. The difference could be due to the different methodology used (such as composition of the medium) between the two studies. Also, these genes may be subjected to additional control by other factors besides PhaR. It has been shown that transcription of these genes in AM1 could be significantly altered upon switching substrates (Good et al., 2015), and other regulators might also control their expression. In *Bradyrhizobium japonicum*, expression of the *phaC* homologous gene bll6073 is controlled by the global regulator FixK_2_ (Mesa et al., 2008), while a defect in the two-component NtrBC regulatory system can result in increased *phaC* expression in *Paracoccus denitrifcans* under certain conditions (Olaya-Abril et al., 2018). Interestingly, a gene (MEXAM1_RS16330) encoding a protein homologous (36% aa identity) to PhaA was up-regulated in Δ*phaR*_CAD compared to WT_CAD (Supplementary Table S10). If this protein confers a similar enzymatic function, this could lead to increased diversion of acetyl-CoA into the EMC pathway and away from the TCA cycle, potentially reducing ITA production.

Of the three genes annotated as depolymerases for PHB mobilization (*depA*, *depB*, and *depC*), only *depB* was down-regulated in Δ*phaR_*CAD (Supplementary Table S10). A study involving AM1 with these three depolymerase genes knocked out did not find major changes in its PHB degradation ability compared to the wildtype, suggesting that other enzymes might be involved in this process (Orita et al., 2014). In our study, we observed an up-regulation of a gene annotated to produce esterase (MEXAM1_RS06580) in Δ*phaR*_CAD. This enzyme was found to be homologous (42% aa identity) to a PHB depolymerase (CNE_RS27970) of *Cupriavidus necator*. Interestingly, a gene (MEXAM1_RS07150) encoding a homolog (30% aa identity) of patatin-like protein PhaZh1 (HFX_6464) in the archaeon *Haloferax mediterranei*, which can be associated with PHA granules and has PHA depolymerase activity (Liu et al., 2015), was up-regulated in Δ*phaR*_CAD when compared to WT_101 (Supplementary Table S10). These two products (MEXAM1_RS06580 and MEXAM1_RS07150) may play a role in PHB mobilization of AM1 and thus contribute to the low PHB content of Δ*phaR*_CAD.

Phasins are proteins which are frequently found to coat PHA granules in bacteria. Phasins are structurally and functionally diverse with roles including furnishing structural stability to PHA granules, PHA depolymerisation, increasing PHA synthase activity, segregation of PHA granules, and chaperone activities (Mezzina and Pettinari, 2016). Korotkova et al. (2002) showed that mutations of two phasin-encoding genes in AM1, *gap11* (MEXAM1_RS10475) and *gap20* (MEXAM1_RS11975), resulted in lower PHB accumulation when grown on methanol, although the exact functions of these phasins in AM1 are currently unknown. They observed no significant change in transcription of these genes in their *phaR* mutant, in contrast to our transcriptome data where both of these phasin-encoding genes were significantly up-regulated in Δ*phaR*_CAD (Supplementary Table S10). Up-regulation of these two phasin-encoding genes suggests that PhaR could be a repressor of expression for the genes encoding phasins, as in *P*. *denitrificans* (Maehara et al., 2002) and *B*. *diazoefficiens* (Quelas et al., 2016; Nishihata et al., 2018). The differences observed between our study and that of Korotkova et al. (2002) implies that other factors might play a role in phasin expression. Nishihata et al. (2018) suggested that the low PHB content in their *B. diazoefficiens* *phaR* mutant strain could be partly attributed to the up-regulation of phasin expression. They speculated that the biosynthesized PHB granules would be immediately covered by the higher concentration of phasins, potentially suppressing further granule enlargement and increasing the activity of PHB depolymerases at the granule surface. Given the up-regulation of *gap11* and *gap20* phasin genes in Δ*phaR*_CAD, the low PHB content observed in this strain may be due to a similar mechanism.

### Transcription of Genes Related to Methanol Metabolism

**FIGURE 6:**
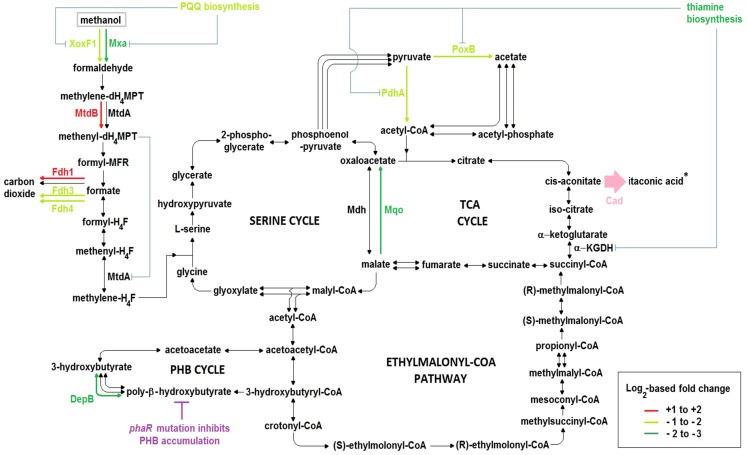
Central carbon metabolic pathways, including key differences in the transcriptomes of Δ*phaR*_CAD versus both WT_101 and WT_CAD. Experiments were performed in the HM medium with 240 mM methanol as the carbon source. Asterisk (^∗^) denotes ITA production by the heterologous Cad (pink color), while the *phaR* mutation causes deficiency in PHB accumulation (magenta color). The color of the arrows in the pathway indicates the level of differential gene expression according to the legend where the + and – signs denote up- and down-regulation, respectively, while black arrows represent genes in pathways that were not differentially expressed. Blue lines indicate possible inhibition or reduction of enzyme activities by a metabolite or as a result of diminished co-factor biosynthesis. PQQ, pyrroloquinoline quinone; XoxF1, lanthanide- and PQQ-dependent methanol dehydrogenase; Mxa, calcium- and PQQ-dependent methanol dehydrogenase; MtdA, methylene-dH_4_MPT/H_4_F dehydrogenase; MtdB, methylene-dH_4_MPT dehydrogenase; Fdh1, tungsten- and NAD-dependent formate dehydrogenase; Fdh3, cytochrome-linked formate dehydrogenase; Fdh4, molybdopterin-binding oxidoreductase-like formate dehydrogenase; DepB, PHB depolymerase; Mdh, NAD-dependent malate dehydrogenase; Mqo, membrane-associated malate:quinone oxidoreductase; PdhA, α-subunit of pyruvate dehydrogenase; PoxB, ubiquinone-dependent pyruvate dehydrogenase; α-KGDH, α-ketoglutarate dehydrogenase complex; Cad, *cis-*aconitic acid decarboxylase.

Consistent with our observations, a previous study has shown that the AM1 *phaR* mutant has reduced biomass yield (25% less) compared to the wildtype when consuming methanol (Van Dien et al., 2003). Here, we observed down-regulation of the biosynthesis operon for the calcium- and PQQ-dependent methanol dehydrogenase (Mxa) in Δ*phaR*_CAD (Figure 6 and Supplementary Table S11). *xoxF1*, encoding a lanthanide- and PQQ-dependent methanol dehydrogenase (Nakagawa et al., 2012), was also down-regulated (Figure 6 and Supplementary Table S11). The down-regulation of these genes was consistent with the slower growth of Δ*phaR*_CAD (Figure 4A). On the other hand, *exaF* was up-regulated in Δ*phaR*_CAD (Supplementary Table S11). ExaF is another PQQ-dependent quinoprotein, which is dependent on lanthanide and functions primarily as an ethanol dehydrogenase but with low catalytic activities toward methanol, formaldehyde and acetaldehyde (Good et al., 2016). Here, it could be that ExaF has a role to play in fine-tuning the overall methanol oxidation pathway.

The *pqqA* gene in the PQQ biosynthesis operon, encoding a peptide precursor of PQQ, was down-regulated in Δ*phaR*_CAD (Supplementary Table S12). Toyama and Lidstrom (Toyama and Lidstrom, 1998) have shown that disruption of this gene could reduce PQQ production in AM1 but does not completely abolish its biosynthesis. Two genes (MEXAM1_RS21885 and MEXAM1_RS21890) in Δ*phaR*_CAD whose products are inferred to be homologs of PqqA were found to be down-regulated (Supplementary Table S12). Some bacteria, such as *Methylovorus* sp. MP688, are known to have multiple copies of *pqqA* that respond to different stimuli (Ge et al., 2015). The two *pqqA* homologous genes in AM1 may produce the PQQ peptide precursor, explaining the continued PQQ production by the *pqqA*-disrupted AM1 mutant strain (Toyama and Lidstrom, 1998). The reduced expression of the three genes above may lower the availability of PQQ, an important co-factor for the activities of both the methanol dehydrogenases Mxa and XoxF1 (Figure 6). This in turn might contribute to lower methanol uptake and thus reduce the carbon flow toward biomass growth and ITA production in Δ*phaR*_CAD.

Two main pathways involving several enzymes are responsible for C-1 transfer in AM1, one relying on H_4_F and the other on dH_4_MPT as the C-1 carrier (Vorholt, 2002). The dH_4_MPT-based pathway participates in formaldehyde oxidation to formate, which forms a branch point as it can either be oxidized to generate reducing power or be assimilated into biomass via the H_4_F-based pathway (Crowther et al., 2008). In our experiments, *mtdB*, which encodes a methylene-dH_4_MPT dehydrogenase that uses either NAD^+^ or NADP^+^ and is involved in the dH_4_MPT-dependent pathway, was up-regulated in Δ*phaR*_CAD (Supplementary Table S11). MtdB is essential for methanol assimilation and also important for formaldehyde detoxification where it converts methylene-dH_4_MPT to methenyl-dH_4_MPT via the dH_4_MPT-based C-1 transfer pathway (Hagemeier et al., 2000), but it cannot dehydrogenate methylene-H_4_F, which is an intermediate in the H_4_F-dependent pathway (Hagemeier et al., 2000). On the other hand, there was no differential expression of the gene encoding MtdA, which catalyzes the oxidation of methylene-dH_4_MPT to methenyl-dH_4_MPT and reduction of methenyl-H_4_F to methylene-H_4_F, but has been suggested to be primarily involved in the H_4_F-dependent pathway (Marx and Lidstrom, 2004). Previous studies have shown that MtdB serves as the main methylene-dH_4_MPT dehydrogenase *in vivo* (Hagemeier et al., 2000; Marx and Lidstrom, 2004), while MtdA has a role in regulating the segregation of C-1 carbon flux between assimilation and oxidation (Martinez-Gomez et al., 2013, 2015). The higher *mtdB* gene expression in Δ*phaR*_CAD might result in more methenyl-dH_4_MPT production from methylene-dH_4_MPT that can then act as a regulatory signal by controlling the enzymatic activity of MtdA via inhibition of the reduction of methenyl-H_4_F to methylene-H_4_F (Martinez-Gomez et al., 2013, 2015) (Figure 6). In essence, up-regulation of the *mtdB* gene might impede biomass accumulation but promote formate oxidation to generate more NADH as energy (Figure 6), explaining the slower growth phenotype observed for Δ*phaR*_CAD (Figure 4A).

Formate is at an important branch point in the central carbon network during methylotrophic metabolism in AM1, as it can either be utilized for biomass production via the serine cycle or for energy generation by formate dehydrogenases (Crowther et al., 2008). AM1 has four known formate dehydrogenases (Fdh1–4) with different co-factor requirements. Fdh1 and Fdh2 are dependent on NAD^+^ for formate oxidation but require tungsten and molybdenum, respectively, while cytochrome-linked Fdh3 and molybdopterin-binding oxidoreductase-like protein Fdh4 do not require NAD^+^ (Chistoserdova et al., 2004, 2007). In Δ*phaR*_CAD, we observed the up-regulation of genes related to Fdh1 (and also Fdh2 when compared to WT_101), while those associated with Fdh3 and Fdh4 were down-regulated (Figure 6 and Supplementary Table S11). Previously, Fdh4 has been implicated in methanol metabolism as mutation of the encoding gene resulted in diminished growth on methanol (Chistoserdova et al., 2007). Thus, down-regulation of genes related to Fdh4 biosynthesis in Δ*phaR*_CAD might contribute to its reduced methanol assimilation (Figure 4B).

### Transcription of Genes Related to Pyruvate Metabolism and the TCA Cycle

Pyruvate and acetate are sources for acetyl-CoA, which in turn is the precursor for ITA production via the TCA cycle. Expression of *pdhA* (and also *pdhB* when compared to WT_101) encoding subunits of the E1 component (i.e., pyruvate dehydrogenase) of the pyruvate dehydrogenase complex was down-regulated in Δ*phaR*_CAD, thus potentially reducing acetyl-CoA formation from pyruvate (Figure 6 and Supplementary Table S11). Down-regulation of *pdhA* and *pdhB* has also been observed in the *phaR* mutant of *B. diazoefficiens* (Nishihata et al., 2018). Also down-regulated in Δ*phaR*_CAD was the *poxB* gene encoding the ubiquinone-dependent pyruvate dehydrogenase (Supplementary Table S11), which converts pyruvate to acetate which can subsequently be converted to acetyl-CoA directly by acetyl-CoA synthetase or via acetyl-phosphate by phosphate acetyltransferase. The down-regulation of *pdhA* and *poxB* might reduce the availability of acetyl-CoA in Δ*phaR*_CAD and contribute to reducing its ITA production (Figure 6).

AM1 has two malate dehydrogenases, the NAD-dependent malate dehydrogenase (Mdh) and the membrane-associated malate:quinone oxidoreductase (Mqo). They have different biochemical characteristics, as Mdh requires NAD^+^ as a coenzyme and catalyzes a reversible reaction, while Mqo uses quinone as an electron acceptor and its catalytic reaction is irreversible. In *C. glutamicum*, which contains these two types of malate dehydrogenases, it has been suggested that Mqo is the main enzyme responsible for oxidizing malate to oxaloacetate and its activity is affected by the carbon source used (Molenaar et al., 2000). In Δ*phaR*_CAD, only the *mqo* gene was significantly down-regulated (Supplementary Table S11), which might result in lower oxaloacetate production, and consequently, a reduced flux through the TCA cycle (Figure 6), again potentially impairing its ITA production.

### Transcription of Genes Related to Thiamine Biosynthesis

A gene cluster related to thiamine biosynthesis was down-regulated in Δ*phaR*_CAD (Supplementary Table S12). Reduced availability of thiamine could have a large impact on metabolism as thiamine serves as a co-factor for enzymes involved in many metabolic pathways including pyruvate dehydrogenases (required by both E1 component (i.e., pyruvate dehydrogenase) of the pyruvate dehydrogenase complex and PoxB) and the TCA cycle (specifically for E1 component (i.e., oxoglutarate decarboxylase) of the α-ketoglutarate dehydrogenase complex) (Du et al., 2011). The paucity of thiamine as a co-factor might result in the α-ketoglutarate dehydrogenase complex exhibiting weaker enzyme activity, which might lower the TCA cycle flux and in turn reduce the ITA production in Δ*phaR*_CAD (Figure 6). Likewise, given the reduced availability of the co-factor, the pyruvate dehydrogenases may have impaired conversion of pyruvate to acetyl-CoA, the precursor for ITA biosynthesis, and consequently reduce ITA production (Figure 6).

### ITA Production in Scaled Up Fed-Batch Bioreactors

**FIGURE 7:**
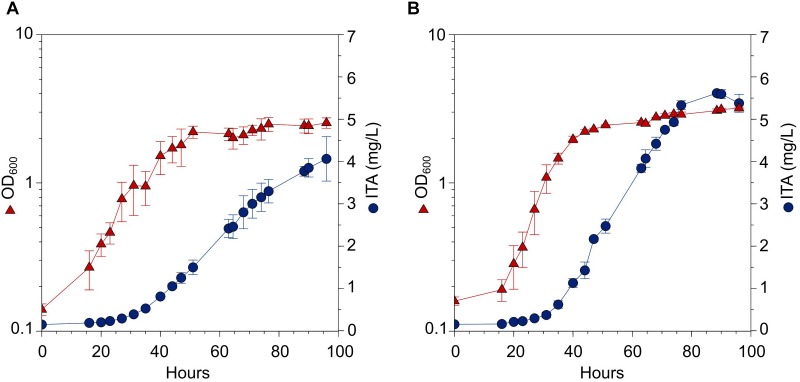
Fed-batch bioreactor experiment of WT_CAD grown in HM medium with methanol as the carbon source. The duration of the fed-batch cultivation was 96 h. **(A)** Dissolved O_2_ saturation level was maintained at 25% and pH control with 1 M NaOH. **(B)** Dissolved O_2_ saturation level was maintained at 60% and pH control with 1 M NH_4_OH. Error bars represent one standard deviation.

Scale-up experiments using fed-batch bioreactors with methanol were performed in an attempt to obtain a higher ITA production titer. Based on the batch cultures, we used the best performing strain WT_CAD for cultivation in a fed-batch bioreactor with dissolved O_2_ saturation level set at 25% and pH maintained by NaOH addition. We obtained a biomass concentration of OD_600_ = 2.5 and ITA titer 4.1 ± 0.5 mg/L, with a productivity of 0.042 ± 0.005 mg/L/h of ITA (Figure 7A). Although the biomass concentration was substantially higher when compared against batch cultures of the same strain provided with methanol and cultured for the same time, the ITA titer achieved from batch culture was higher (i.e., 5.1 ± 0.4 mg/L ITA) (Figure 4D).

Transcriptomic analysis in this study observed lower expression of genes related to cytochrome *o* ubiquinol oxidase system in Δ*phaR*_CAD (Supplementary Table S13). Also, genes linked to oxidative stress response such as catalases and superoxide dismutase were down-regulated, suggesting a lower metabolic respiration in Δ*phaR*_CAD (Supplementary Table S13). This is supported by Strovas et al. (2006) who previously determined that AM1 *phaR* mutant strain has reduced oxygen consumption rate when using methanol as the carbon source. Based on this finding, we suspected that dissolved oxygen concentration could be a factor controlling ITA production in the engineered strain, as has been shown in *E. coli* (Chang et al., 2017). Accordingly, we performed another fed-batch bioreactor experiment with a relative higher dissolved O_2_ level of 60% and used NH_4_OH to control the pH (instead of NaOH), which also provided a source of nitrogen. Under these conditions, we obtained a higher biomass concentration (OD_600_ = 3.2) and slight improvement in ITA titer (5.4 ± 0.2 mg/L) and productivity (0.056 ± 0.002 mg/L/h) compared to the 25% dissolved O_2_ experiment (Figure 7B). Despite the higher cell density, the ITA titer obtained was similar to that achieved using WT_CAD in batch cultures provided with methanol after the same cultivation period of 96 h (i.e., 5.1 ± 0.4 mg/L ITA) (Figure 4D). However, the accumulation of ITA plateaued at a later stage in the bioreactors. This observation suggests that in the bioreactor cultures, more of the carbon was used for biomass formation or energy generation instead of being channeled toward ITA production. Further investigation is required to determine the exact metabolic mechanisms behind the observed phenotypes.

## Conclusion

In this study, we successfully engineered AM1 to produce ITA. We tried to enhance ITA production by introducing a *phaR* mutation, but this resulted in lower production than from the ITA-producing engineered wildtype strain. RNA-Seq analysis elucidated possible reasons for this unexpected result, with generally higher expression of pathways that might divert carbon flux away from ITA biosynthesis. This study provided evidence that PhaR might have a broader regulatory role than previously anticipated, and further research on how to best engineer methylotrophic bacteria for ITA production is required. Our transcriptomic results suggest some genes of interest for potential improvements in AM1 ITA production, including *fdh1AB*, *mtdB*, *mqo*, *pdhAB*, and *poxB*. Future works should consider constructing strains with these genes overexpressed or suppressed, in conjunction with metabolite profiling. Our RNA-Seq analysis also provides hints that the genes encoding proteins homologous to PhaA, PHB depolymerase, and patatin-like protein PhaZh1 might affect ITA production and future investigations should characterize the activities of these gene products. In addition, our results demonstrated the sensitivity of AM1 ITA production to culture conditions, suggesting further process engineering is required to optimize ITA production in scaled-up bioreactors.

## Author Contributions

CL and PL conceived and designed the study. CL, PL, and JV analyzed the data and wrote the manuscript. HL contributed to some initial works. CL, AC, and MD performed the experiments. All the authors read and approved the final manuscript.

## Conflict of Interest Statement

The authors declare that the research was conducted in the absence of any commercial or financial relationships that could be construed as a potential conflict of interest.

## Abbreviations

aa: amino acids
Ap^R^: ampicillin resistance
cDNA: complementary DNA
CM: minimal media adapted from Choi et al. (1989)
Cm^R^: chloramphenicol resistance
dH_4_MPT: dephosphotetrahydromethanopterin
EMC: ethylmalonyl-CoA
Gb: gigabyte
gDNA: genomic DNA
H_4_F: tetrahydrofolate
HM: minimal media adapted from Mokhtari-Hosseini et al. (2009)
Km^R^: kanamycin resistance
MC: minimal media adapted from Zhu et al. (2016)
MM: ATCC 1057 Methylococcus medium
mRNA: messenger RNA
OD_600_: optical density at 600 nm wavelength
PCR: polymerase chain reaction
PHA: polyhydroxyalkanoate
PHB: poly-β-hydroxybutyric acid
PQQ: pyrroloquinoline quinone
RNA-Seq: RNA sequencing
rRNA: ribosomal RNA
sRNA: small RNA
Str^R^: streptomycin resistance
TCA: tricarboxylic acid
Tc^R^: tetracycline resistance
