# Supplementary material for: Designing and Engineering Methylorubrum extorquens AM1 for Itaconic Acid Production
Source: Front Microbiol. 2019 May 9;10:1027. doi: 10.3389/fmicb.2019.01027 (PMC6520949; doi:10.3389/fmicb.2019.01027)
Supplement: Supplementary file 1 [file Data_Sheet_1.docx]

**Supplementary material 1**

**A**

**
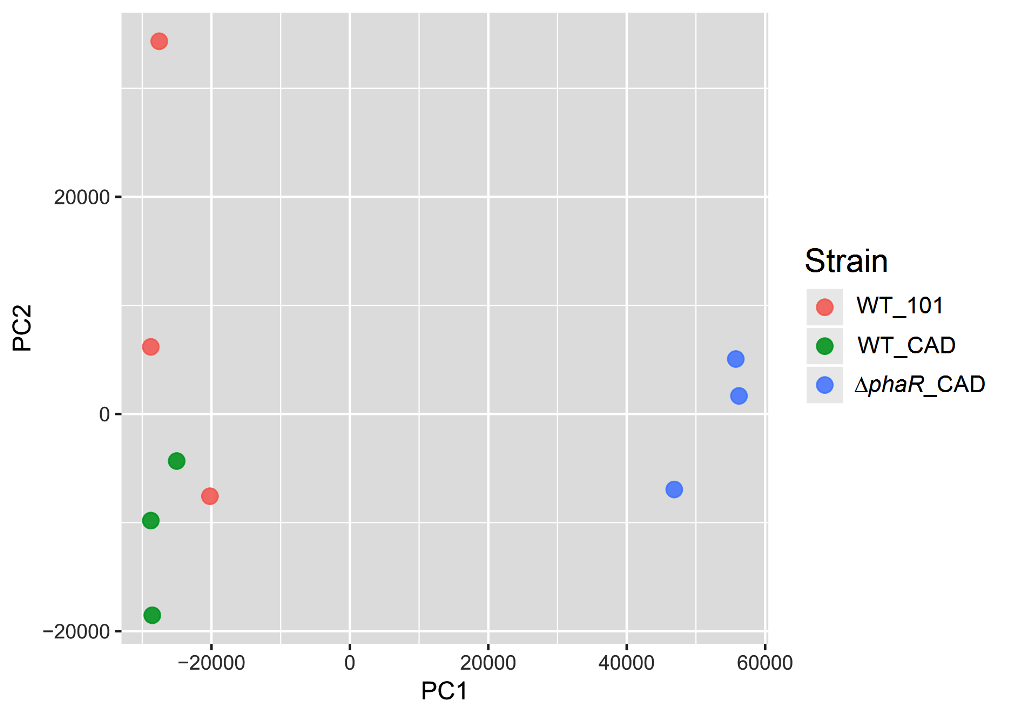
**

**B**

**
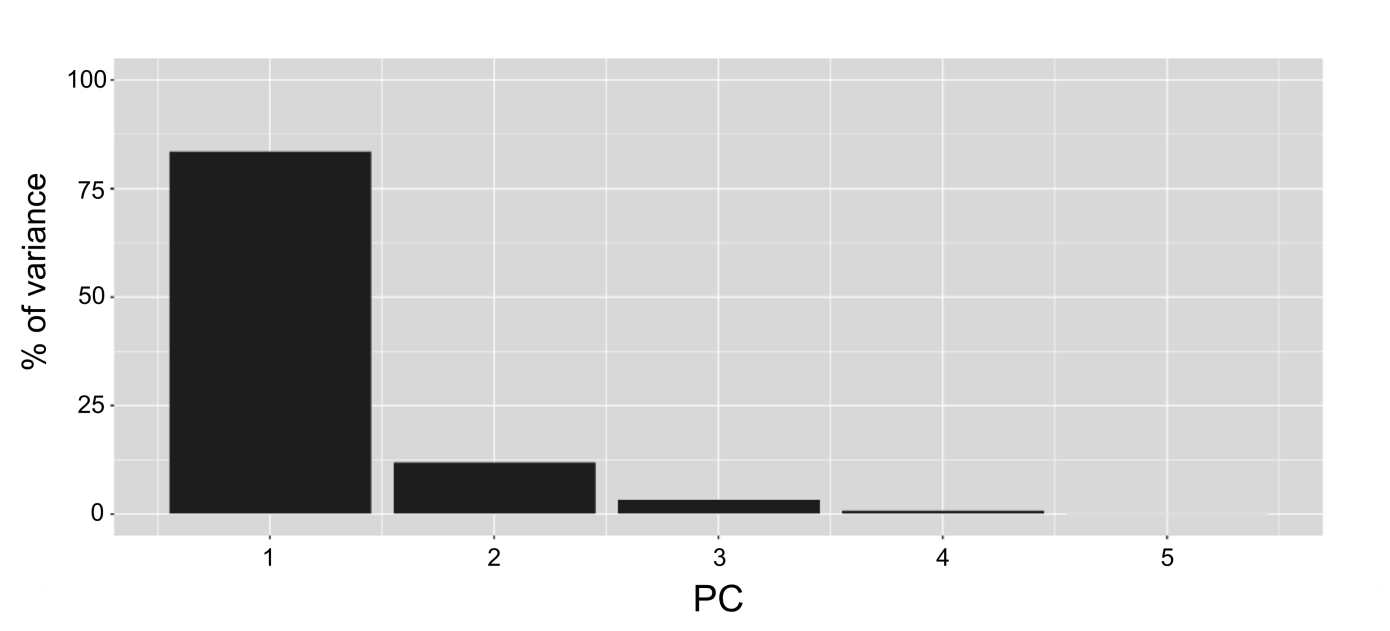
**

**Figure S1. (A)** Principal component analysis of the transcriptomic data of each strain. Each data point represents a biological sample. **(B)** The percent variation explained by each principal component (PC).


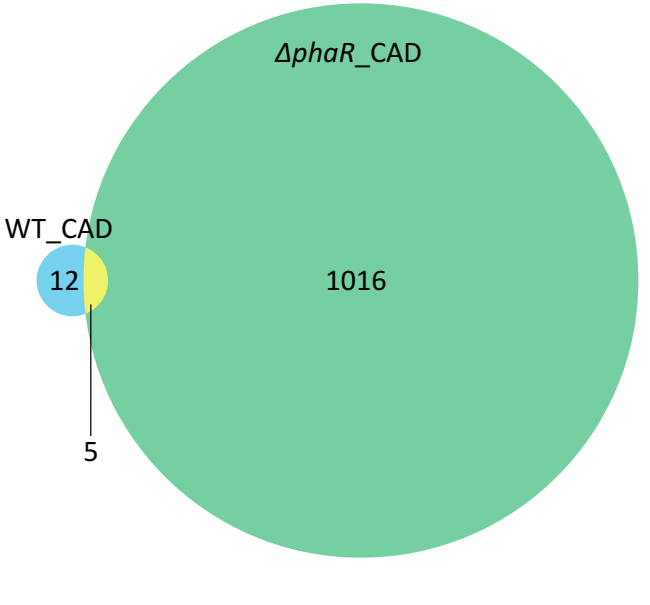


**Figure S2.** A Venn diagram showing the number of shared differentially expressed genes between WT_CAD and Δ*phaR*_CAD relative to WT_101.

**Table S1**. Composition of minimal media used in this study

1. MC medium, adapted from [Zhu et al. (2016](#_ENREF_12)) with minor modifications

| **Solution** | **Preparation^†^** | **Final concentration** |
| --- | --- | --- |
| **X** | In 499 mL H_2_O |  |
| (NH_4_)_2_SO_4_ | 1 g | 1 g/L |
| MgSO_4_^.^7H_2_O | 0.449 g | 0.449 g/L |
| CaCl_2_^.^2H_2_O | 0.0032 g | 0.0032 g/L |
|  |  |  |
| **Y** | In 50 mL H_2_O |  |
| Na_3_C_6_H_3_O_7_^.^2H_2_O | 0.671 g | 0.01342 g/L |
| ZnSO_4_^.^7H_2_O | 0.0173 g | 0.0146 g/L |
| MnCl_2_^.^4H_2_O | 0.0099 g | 0.198 mg/L |
| FeSO_4_^.^7H_2_O | 0.25 g | 0.005 g/L |
| Na_2_MoO_4_^.^2H_2_O | 0.0242 g | 0.484 mg/L |
| CuCl_2_^.^2H_2_O | 0.0085 g | 0.17 mg/L |
| CoCl_2_^.^6H_2_O | 0.0238 g | 0.476 mg/L |
| Na_2_WO_4_^.^2H_2_O | 0.0054 g | 0.108 mg/L |
| H_3_BO_3_ | 0.0015 g | 0.03 mg/L |
|  |  |  |
| **Z** | In 500 mL H_2_O |  |
| KH_2_PO_4_ | 1.3051 g | 1.3051 g/L |
| Na_2_HPO_4_^.^2H_2_O | 2.6699 g | 2.6699 g/L |

^†^ 499 mL of X was mixed with 1 mL of Y and autoclaved, while 500 mL of Z was adjusted to pH 6.8 and autoclaved. After cooling, 500 mL of X+Y was combined with 500 mL of Z.

1. CM medium, adapted from [Choi et al. (1989](#_ENREF_3)) with minor modifications

| **Solution** | **Preparation^†^** | **Final concentration** |
| --- | --- | --- |
| **K** | In 499 mL H_2_O |  |
| (NH_4_)_2_SO_4_ | 1. g | 1 g/L |
| MgSO_4_^.^7H_2_O | 0.45 g | 0.45 g/L |
| CaCl_2_^.^2H_2_O | 0.0033 g | 3.3 mg/L |
| FeSO_4_^.^7H_2_O | 0.0013 g | 1.3 mg/L |
|  |  |  |
| **L** | In 50 mL H_2_O |  |
| MnCl^.^4H_2_O | 0.005 g | 0.1 mg/L |
| Na_2_MoO_4_^.^2H_2_O | 0.002 g | 40 μg/L |
| CuCl_2_^.^2H_2_O | 0.0014 g | 27 μg/L |
| CoCl_2_^.^6H_2_O | 0.002 g | 40 μg/L |
| ZnSO_4_^.^7H_2_O | 0.0065 g | 130 μg/L |
|  |  |  |
| **M** | In 500 mL H_2_O |  |
| KH_2_PO_4_ | 1.305 g | 1.305 g/L |
| Na_2_HPO_4_^.^2H_2_O | 2.70 g | 2.70 g/L |

^†^ 499 mL of K was mixed with 1 mL of L and autoclaved, while 500 mL of M was adjusted to pH 6.8 and autoclaved. After cooling, 500 mL of K+L was combined with 500 mL of M.

1. HM medium, adapted from [Mokhtari-Hosseini et al. (2009](#_ENREF_6)) with minor modifications

| **Solution** | **Preparation^†^** | **Final concentration** |
| --- | --- | --- |
| **A** | In 498 mL H_2_O |  |
| (NH_4_)_2_SO4 | 1.75 g | 1.75 g/L |
| MgSO_4_^.^7H_2_O | 0.1 g | 0.1 g /L |
| FeSO_4_^.^7H_2_O | 0.02 g | 20 mg/L |
| CaCl_2_^.^2H_2_O | 0.02 g | 20 mg/L |
|  |  |  |
| **B** | In 100 mL H_2_O |  |
| Na_2_MoO_4_^.^2H_2_O | 0.004 g | 0.04 mg/L |
| CuCl_2_^.^2H_2_O | 0.0027 g | 0.027 mg/L |
|  |  |  |
| **C** | In 50 mL H_2_O |  |
| MnCl_2_^.^4H_2_0 | 0.18 g | 3.6 mg/L |
| ZnSO_4_^.^7H_2_O | 0.075 g | 1.5 mg/L |
| CoCl_2_^.^6H_2_O | 0.03 g | 0.6 mg/L |
| H_3_BO_3_ | 0.01 g | 0.2 mg/L |
|  |  |  |
| **D** | In 500 mL H_2_O |  |
| KH_2_PO_4_ | 0.68 g | 0.68 g/L |
| Na_2_HPO_4_^.^2H_2_O | 3.051 g | 3.05 g/L |

^†^ 498 mL of A was mixed with 1 mL each of B and C. This mixture was autoclaved, while 500 mL of D was adjusted to pH 7.0 prior to autoclaving. After cooling, 500 mL of A+B+C was combined with 500 mL of D.

1. MM medium, adapted from ATCC 1057 medium with minor modifications

| **Solution** | **Preparation^†^** | **Final concentration** |
| --- | --- | --- |
| **E** | In 983.5 mL H_2_O |  |
| NaNO_3_ | 2.0 g | 0.002 g/L |
|  |  |  |
| **F** | In 100 mL H_2_O |  |
| KH_2_PO_4_ | 13.6 g | 0.884 g/L |
| NaOH | 2.88 g | 0.1872 g/L |
|  |  |  |
| **G** | In 1000 mL H_2_O |  |
| MgSO_4_^.^7H_2_O | 20.0 g | 0.2 g/L |
| CaCl_2_^.^2H_2_O | 2.0 g | 0.02 g/L |
| ZnSO_4_^.^7H_2_O | 0.049 g | 0.49 mg/L |
| CuCl_2_^.^2H_2_O | 0.002 g | 0.02 mg/L |
| H_3_BO_3_ | 0.006 g | 0.06 mg/L |
| MnCl_2_^.^4H_2_O | 0.0027 g | 0.027 mg/L |
| FeSO_4_^.^7H_2_O | 1.0 g | 0.01 g/L |

^†^ 983.5 mL of E was mixed with 10 mL of G and autoclaved, while 100 mL of F was adjusted to pH 7.1 and autoclaved. After cooling, 993.5 mL of E+G was combined with 6.5 mL of F.

**Table S2.** Primers used in this study

| **Primers for creating *phaR* in-frame truncation** | **Sequence** |
| --- | --- |
| phaR_Up-F | 5’-NNNGGATCCTCCGCACCACATCGAC-3’ |
| phaR_Up-R | 5’-NNNCTGCAGAGTAGGGCCTCGTCCC-3’ |
| phaR_Down-F | 5’-NNNCTGCAGCGTAGAAGCGGATCAGCT-3’ |
| phaR_Down-R | 5’-NNNATTAATCGATATCTTCACTGGCTGCC-3’ |

**Table S4.** Codon-optimized *cad* gene sequence used in study

| **Amino acid sequence of *cis*-aconitic acid decarboxylase from *Aspergillus terreus* (BAG49047.1)** |
| --- |
| MTKQSADSNAKSGVTSEICHWASNLATDDIPSDVLERAKYLILDGIACAWVGARVPWSEKYVQATMSFEPPGACRVIGYGQKLGPVAAAMTNSAFIQATELDDYHSEAPLHSASIVLPAVFAASEVLAEQGKTISGIDVILAAIVGFESGPRIGKAIYGSDLLNNGWHCGAVYGAPAGALATGKLLGLTPDSMEDALGIACTQACGLMSAQYGGMVKRVQHGFAARNGLLGGLLAHGGYEAMKGVLERSYGGFLKMFTKGNGREPPYKEEEVVAGLGSFWHTFTIRIKLYACCGLVHGPVEAIENLQGRYPELLNRANLSNIRHVHVQLSTASNSHCGWIPEERPISSIAGQMSVAYILAVQLVDQQCLLSQFSEFDDNLERPEVWDLARKVTSSQSEEFDQDGNCLSAGRVRIEFNDGSSITESVEKPLGVKEPMPNERILHKYRTLAGSVTDESRVKEIEDLVLGLDRLTDISPLLELLNCPVKSPLV |
| **Codon-optimized *cad* gene sequence used in study (5’ 🡪 3’)** |
| ATGACCAAGCAGTCCGCCGATTCGAACGCCAAGTCGGGCGTCACGAGCGAGATCTGCCACTGGGCCTCGAACCTGGCCACCGACGATATCCCCTCCGACGTGCTGGAACGCGCGAAGTACCTGATCCTTGACGGCATCGCCTGCGCCTGGGTCGGCGCACGAGTGCCCTGGAGCGAGAAGTACGTCCAGGCAACGATGAGCTTCGAGCCGCCGGGCGCGTGCCGCGTCATCGGGTACGGCCAGAAGCTGGGGCCGGTGGCCGCCGCTATGACGAACTCGGCCTTCATCCAGGCGACCGAGCTCGACGACTACCACTCCGAAGCACCGCTGCATTCCGCCTCGATCGTCCTCCCGGCCGTCTTCGCGGCCAGTGAGGTGTTGGCCGAGCAGGGCAAGACCATCTCCGGCATCGACGTGATCCTCGCTGCGATCGTAGGCTTCGAGTCCGGCCCCAGGATCGGCAAGGCCATCTACGGCTCGGACCTCCTGAACAACGGCTGGCACTGCGGGGCCGTCTACGGTGCCCCGGCGGGCGCCCTGGCGACGGGCAAGCTCCTCGGCCTGACGCCGGACTCCATGGAAGATGCCTTAGGTATCGCCTGCACCCAGGCCTGCGGCCTGATGTCGGCGCAGTACGGCGGCATGGTCAAGCGCGTCCAGCACGGGTTCGCCGCTAGGAACGGGTTGCTTGGCGGCCTCCTGGCCCACGGTGGTTACGAGGCGATGAAGGGCGTGCTGGAGCGCTCTTACGGCGGCTTCCTGAAGATGTTCACCAAGGGCAACGGACGGGAGCCGCCCTACAAAGAGGAAGAGGTCGTCGCGGGCCTGGGCAGCTTCTGGCACACCTTCACGATTCGGATCAAGCTCTATGCGTGCTGCGGCTTGGTGCACGGTCCGGTTGAGGCCATCGAGAACCTCCAGGGCCGCTACCCGGAACTCCTGAACCGCGCCAACCTGTCCAACATCCGGCATGTCCACGTCCAGCTGAGCACCGCCTCGAACTCGCATTGCGGCTGGATCCCCGAGGAGCGTCCGATCTCGTCCATTGCCGGCCAGATGTCGGTGGCGTATATCCTGGCGGTGCAGCTCGTCGACCAGCAGTGCCTGCTCAGCCAGTTCTCGGAGTTCGATGACAACCTTGAGCGGCCTGAAGTCTGGGACCTCGCCCGCAAGGTCACCTCCTCGCAGAGCGAGGAGTTCGACCAGGACGGCAACTGCCTCAGCGCAGGCCGCGTGAGGATCGAGTTCAACGACGGCTCCTCCATCACGGAATCGGTCGAGAAGCCCCTGGGCGTCAAGGAGCCGATGCCGAACGAGCGCATCCTGCACAAGTACCGCACGCTGGCGGGTTCGGTCACGGACGAGTCCCGCGTCAAGGAGATCGAGGATCTCGTGCTGGGCCTGGATCGCCTCACCGACATCTCACCGCTTCTCGAGCTGCTCAACTGCCCGGTCAAGAGCCCGCTGGTGTGA |

**Table S5.** Growth rate and doubling time of the strains

| **Strain**^a^ | **Growth rate** (h^-1^)^b^ | **Doubling time** (h)^c^ |
| --- | --- | --- |
| WT_101 | 0.0753 ± 0.004 | 9.31 ± 0.56 |
| WT_CAD | 0.0760 ± 0.002 | 9.12 ± 0.27 |
| Δ*phaR*_CAD | 0.0593 ± 0.007 | 11.8 ± 1.25 |

^a^ Strains were grown as batch culture in the HM medium with 240 mM methanol as the carbon substrate

^b, c^ The growth rate and doubling time were calculated based on the initial 2-day period during exponential growth

**Table S6.** Selected examples of bacterial hosts engineered to produce ITA

| **Organism** | **ITA titer**  (g/L) | **ITA productivity**  (g/L/h) | **ITA yield**  (mol/mol carbon in substrate) | **Cultivation method** | **Carbon substrate** | **Reference** |
| --- | --- | --- | --- | --- | --- | --- |
| *C. glutamicum* | 7.8 | 0.27 | 0.067 | Shake-flask cultivation | Glucose | Otten et al. ([2015](#_ENREF_10)) |
| *E. coli* | 0.69 | 0.01 | 0.015 | Batch bioreactor | Glucose | Vuoristo et al. ([2015](#_ENREF_11)) |
| *E. coli* | 4.3 | 0.04 | Not available | Batch bioreactor | Glucose | Okamoto et al. ([2014](#_ENREF_8)) |
| *E. coli* | 0.86 | 0.012 | 0.023 | Shake-flask cultivation | Glycerol | Jeon et al. ([2016](#_ENREF_4)) |
| *E. coli* | 42 | 2.2 | 0.11 | Shake-flask cultivation | Citrate | Kim et al. ([2017](#_ENREF_5)) |
| *E. coli* | 3.6 | 0.07 | 0.027 | Fed-batch bioreactor | Acetate | Noh et al. ([2018](#_ENREF_7)) |
| *E. coli* | 43 | 1.3 | 0.14 | Fed-batch bioreactor | Glycerol | Chang et al. ([2017](#_ENREF_1)) |
| *E. coli* | 20 | 0.27 | 0.11 | Shake-flask cultivation | Xylose | Chang et al. ([2017](#_ENREF_1)) |
| *E. coli* | 0.15 | 0.002 | Not available | Batch bioreactor | Starch | Okamoto et al. ([2015](#_ENREF_9)) |
| *Synechocystis* sp. | 0.015 | 3.8 × 10^-5^ | Not available | Fed-batch bioreactor | Carbon dioxide | Chin *et al*. ([2015](#_ENREF_2)) |
| *M. extorquens* AM1 (WT_CAD) | 0.032 | 1.3 × 10^-4^ | 0.020 | Shake-flask  cultivation | Methanol | This study |

**Table S9.** Differential expression of selected genes of interest related to proteins with regulatory roles

| **Locus tag**  (MEXAM1_ ) | **Encoded product** | **Log_2_-based fold change^†^** | | |
| --- | --- | --- | --- | --- |
|  |  | **WT_CAD vs.**  **WT_101** | **Δ*phaR*_CAD vs.**  **WT_101** | **Δ*phaR*_CAD vs.**  **WT_CAD** |
| RS14660 | Phyllosphere-induced regulator, PhyR | -0.119 | **-1.020** | -0.901 |
| RS06090 | Hypothetical protein, NepR homolog | 0.613 | **1.862** | **1.249** |
| RS28640 | Hypothetical protein, NepR homolog | 0.039 | **-2.643** | **-2.681** |
| RS14255 | RNA-binding protein Hfq | 0.154 | **1.451** | **1.297** |
| RS24770 | GNAT family N-acetyltransferase, protein acetyltransferase (Pat) homolog | 0.206 | -0.941 | **-1.147** |

^†^ Bolded numbers denote significant differential gene expression

**Table S10.** Differential expression of selected genes of interest related to PHB metabolism

| **Locus tag**  (MEXAM1_ ) | **Encoded product** | **Log_2_-based fold change^†^** | | |
| --- | --- | --- | --- | --- |
|  |  | **WT_CAD vs.**  **WT_101** | **Δ*phaR*_CAD vs.**  **WT_101** | **Δ*phaR*_CAD vs. WT_CAD** |
| RS16330 | Acetyl-CoA acetyltransferase, PhaA homolog | -0.352 | 0.660 | **1.011** |
| RS21730 | Polyhydroxyalkanoate depolymerase, DepB | 0.050 | **-2.173** | **-2.223** |
| RS06580 | Esterase, PHB depolymerase homolog | -0.208 | **1.195** | **1.403** |
| RS07150 | Esterase,  patatin-like protein PhaZh1 (HFX_6464) homolog | 0.197 | **1.054** | 0.857 |
| RS10475 | Phasin, Gap11 | -0.648 | **2.830** | **3.479** |
| RS11975 | Phasin, Gap20 | -0.129 | **2.158** | **2.287** |

^†^ Bolded numbers denote significant differential gene expression

**Table S11.** Differential expression of selected genes of interest related to carbon metabolism

| **Locus tag**  (MEXAM1_ ) | **Encoded product** | **Log_2_-based fold change^†^** | | |
| --- | --- | --- | --- | --- |
|  |  | **WT_CAD vs.**  **WT_101** | **Δ*phaR*_CAD vs.**  **WT_101** | **Δ*phaR*_CAD vs. WT_CAD** |
| Methanol de-hydrogenase |  |  |  |  |
| RS21365 | Hypothetical protein | 0.153 | **-1.701** | **-1.854** |
| RS21370 | DNA-binding response regulator, MxaB | -0.374 | **-1.194** | -0.819 |
| RS21375 | Hypothetical protein, MxaH | 0.230 | **-1.675** | **-1.905** |
| RS21380 | YncE family protein, MxaE | -0.565 | **-2.619** | **-2.054** |
| RS21385 | SRPBCC family protein, MxaD | -0.348 | **-2.017** | **-1.669** |
| RS21390 | VWA domain-containing protein, MxaL | -0.321 | **-2.297** | **-1.976** |
| RS21395 | hypothetical protein, MxaK | 0.261 | **-1.206** | **-1.468** |
| RS21400 | VWA domain-containing protein, MxaC | -0.320 | **-2.650** | **-2.330** |
| RS21405 | Hypothetical protein, MxaA | -0.095 | **-2.186** | **-2.091** |
| RS21410 | MxaS protein, involved in methanol oxidation | -0.051 | **-2.590** | **-2.539** |
| RS21415 | protein MoxR | -0.334 | **-2.739** | **-2.405** |
| RS21420 | Methanol dehydrogenase [cytochrome c] subunit 2, MxaI | -0.271 | **-2.848** | **-2.578** |
| RS21425 | Cytochrome c-L, MxaG | -0.333 | **-2.070** | **-1.737** |
| RS21430 | Possible chaperone protein, MxaJ | -0.300 | **-2.093** | **-1.793** |
| RS21435 | Methanol dehydrogenase [cytochrome c] subunit 1, MxaF | -0.264 | **-1.644** | **-1.381** |
| Lanthanides-dependent enzymes |  |  |  |  |
| RS08325 | PQQ-dependent dehydrogenase, methanol/ethanol family XoxF1 | -0.162 | **-1.263** | **-1.101** |
| RS05380 | PQQ-dependent dehydrogenase, methanol/ethanol family, ExaF | -0.187 | **1.394** | **1.581** |
| C-1 transfer pathway |  |  |  |  |
| RS08425 | NAD(P)-dependent methylene-dH_4_MPT dehydrogenase, MtdB | -0.129 | **1.238** | **1.368** |
| Formate oxidation |  |  |  |  |
| RS23810 | NADH-quinone oxidoreductase subunit F, tungsten-dependent formate DH, β subunit, Fdh1B | 0.109 | **1.875** | **1.765** |
| RS23815 | Formate dehydrogenase subunit a, tungsten-dependent formate DH, α subunit, Fdh1A | 0.377 | **1.731** | **1.354** |
| RS22905 | NADH-quinone oxidoreductase subunit E, molybdenum-dependent formate DH, γ subunit, Fdh2C | **1.050** | 0.835 | -0.215 |
| RS22920 | Peptidase, molybdenum-dependent formate DH, δ subunit, Fdh2D | 0.682 | **1.020** | 0.338 |
| RS01435 | Formate dehydrogenase, possibly related to Fdh3 biosynthesis | 0.270 | **-1.343** | **-1.613** |
| RS01445 | 4Fe-4S dicluster domain-containing protein, Cytochrome-linked formate DH, Fdh3B | -0.048 | **-1.173** | **-1.125** |
| RS09885 | Hypothetical protein, formate dehydrogenase subunit B, Fdh4B | -0.357 | **-1.440** | **-1.083** |
| RS09890 | Oxidoreductase, formate dehydrogenase subunit A, Fdh4A | -0.203 | **-1.180** | -0.977 |
| Pyruvate metabolism |  |  |  |  |
| RS14075 | Pyruvate dehydrogenase (acetyl-transferring) E1 component subunit α, PdhA | -0.081 | **-1.084** | **-1.004** |
| RS14080 | Pyruvate dehydrogenase complex E1 component subunit β, PdhB | -0.152 | **-1.146** | -0.994 |
| RS06970 | Ubiquinone-dependent pyruvate dehydrogenase, PoxB | 0.145 | **-1.819** | **-1.964** |
| TCA cycle |  |  |  |  |
| RS06705 | Malate dehydrogenase (quinone), Mqo | 0.029 | **-2.547** | **-2.576** |

^†^ Bolded numbers denote significant differential gene expression

**Table S12.** Differential expression of selected genes of interest related to co-factor biosynthesis

| **Locus tag**  (MEXAM1_ ) | **Encoded product** | **Log_2_-based fold change^†^** | | |
| --- | --- | --- | --- | --- |
|  |  | **WT_CAD**  **vs.**  **WT_101** | **Δ*phaR*_CAD**  **vs.**  **WT_101** | **Δ*phaR*_CAD vs. WT_CAD** |
| PQQ biosynthesis |  |  |  |  |
| RS08380 | Pyrroloquinoline quinone precursor peptide, PqqA | -0.109 | **-1.396** | **-1.287** |
| RS21885 | Pyrroloquinoline quinone precursor peptide, PqqA homolog | -0.194 | **-1.209** | **-1.016** |
| RS21890 | Pyrroloquinoline quinone precursor peptide, PqqA homolog | -0.251 | **-1.468** | **-1.218** |
| Thiamine biosynthesis |  |  |  |  |
| RS02050 | Thiamine phosphate synthase | -0.028 | **-2.151** | **-2.123** |
| RS02055 | Thiazole synthase | 0.057 | **-1.560** | **-1.617** |
| RS02060 | Thiamine biosynthesis protein, ThiS | 0.302 | **-1.531** | **-1.833** |
| RS02065 | Glycine oxidase, ThiO | 0.190 | -0.972 | **-1.162** |

^†^ Bolded numbers denote significant differential gene expression

**Table S13.** Differential expression of selected genes of interest related to a terminal oxidase and oxidative stress response

| **Locus tag**  (MEXAM1_ ) | **Encoded product** | **Log_2_-based fold change^†^** | | | |
| --- | --- | --- | --- | --- | --- |
|  |  | **WT_CAD**  **vs.**  **WT_101** | | **Δ*phaR*_CAD**  **vs.**  **WT_101** | **Δ*phaR*_CAD vs. WT_CAD** |
| Cytochrome *o* ubiquinol oxidase |  |  |  | |  |
| RS22380 | Cytochrome *o* ubiquinol oxidase subunit IV, CyoD | -0.017 | **-1.216** | | **-1.199** |
| RS22390 | Cytochrome *o* ubiquinol oxidase subunit I, CyoB | 0.026 | **-1.010** | | **-1.036** |
| RS22395 | Cytochrome *o* ubiquinol oxidase subunit II, CyoA | 0.068 | -0.939 | | **-1.007** |
| Oxidative stress response |  |  |  | |  |
| RS00840 | Catalase | 0.302 | **-1.872** | | **-2.174** |
| RS03085 | Catalase | -0.094 | **-1.027** | | -0.933 |
| RS14005 | Catalase | -0.088 | **-2.895** | | **-2.807** |
| RS21830 | Manganese catalase | 0.011 | **-1.243** | | **-1.254** |
| RS15555 | Hypothetical protein, catalase homolog | -0.505 | **-1.493** | | -0.988 |
| RS16610 | Superoxide dismutase | -0.142 | **-1.221** | | **-1.080** |

^†^ Bolded numbers denote significant differential gene expression

**References**

Chang, P., Chen, G.S., Chu, H.Y., Lu, K.W., and Shen, C.R. (2017). Engineering efficient production of itaconic acid from diverse substrates in *Escherichia coli*. *J Biotechnol* 249**,** 73-81. doi: 10.1016/j.jbiotec.2017.03.026.

Chin, T., Sano, M., Takahashi, T., Ohara, H., and Aso, Y. (2015). Photosynthetic production of itaconic acid in *Synechocystis* sp. PCC6803. *J Biotechnol* 195**,** 43-45. doi: 10.1016/j.jbiotec.2014.12.016.

Choi, J.H., Kim, J.H., and Jm, L. (1989). Optimization of growth medium and poly-β-hydroxybutyric acid production from methanol in *Methylobacterium organophilum*. *Kor J Appl Microbiol Bioeng* 17**,** 392-396.

Jeon, H.G., Cheong, D.E., Han, Y., Song, J.J., and Choi, J.H. (2016). Itaconic acid production from glycerol using *Escherichia coli* harboring a random synonymous codon-substituted 5'-coding region variant of the *cadA* gene. *Biotechnol Bioeng* 113**,** 1504-1510. doi: 10.1002/bit.25914.

Kim, J., Seo, H.M., Bhatia, S.K., Song, H.S., Kim, J.H., Jeon, J.M., Choi, K.Y., Kim, W., Yoon, J.J., Kim, Y.G., and Yang, Y.H. (2017). Production of itaconate by whole-cell bioconversion of citrate mediated by expression of multiple cis-aconitate decarboxylase (*cadA*) genes in *Escherichia coli*. *Sci Rep* 7**,** 39768. doi: 10.1038/srep39768.

Mokhtari-Hosseini, Z.B., Vasheghani-Farahani, E., Heidarzadeh-Vazifekhoran, A., Shojaosadati, S.A., Karimzadeh, R., and Khosravi Darani, K. (2009). Statistical media optimization for growth and PHB production from methanol by a methylotrophic bacterium. *Bioresour Technol* 100**,** 2436-2443. doi: 10.1016/j.biortech.2008.11.024.

Noh, M.H., Lim, H.G., Woo, S.H., Song, J., and Jung, G.Y. (2018). Production of itaconic acid from acetate by engineering acid-tolerant *Escherichia coli* W. *Biotechnol Bioeng* 115**,** 729-738. doi: 10.1002/bit.26508.

Okamoto, S., Chin, T., Hiratsuka, K., Aso, Y., Tanaka, Y., Takahashi, T., and Ohara, H. (2014). Production of itaconic acid using metabolically engineered *Escherichia coli*. *J Gen Appl Microbiol* 60**,** 191-197. doi: 10.2323/jgam.60.191

Okamoto, S., Chin, T., Nagata, K., Takahashi, T., Ohara, H., and Aso, Y. (2015). Production of itaconic acid in *Escherichia coli* expressing recombinant α-amylase using starch as substrate. *J Biosci Bioeng* 119**,** 548-553. doi: 10.1016/j.jbiosc.2014.10.021.

Otten, A., Brocker, M., and Bott, M. (2015). Metabolic engineering of *Corynebacterium glutamicum* for the production of itaconate. *Metab Eng* 30**,** 156-165. doi: 10.1016/j.ymben.2015.06.003.

Vuoristo, K.S., Mars, A.E., Sangra, J.V., Springer, J., Eggink, G., Sanders, J.P., and Weusthuis, R.A. (2015). Metabolic engineering of itaconate production in *Escherichia coli*. *Appl Microbiol Biotechnol* 99**,** 221-228. doi: 10.1007/s00253-014-6092-x.

Zhu, W.L., Cui, J.Y., Cui, L.Y., Liang, W.F., Yang, S., Zhang, C., and Xing, X.H. (2016). Bioconversion of methanol to value-added mevalonate by engineered *Methylobacterium extorquens* AM1 containing an optimized mevalonate pathway. *Appl Microbiol Biotechnol* 100**,** 2171-2182. doi: 10.1007/s00253-015-7078-z.
